# A Retrospective Analysis of Biological Complications of Dental Implants

**DOI:** 10.1155/2022/1545748

**Published:** 2022-08-12

**Authors:** Momen A. Atieh, Zainab Almutairi, Fatemeh Amir-Rad, Mohammed Koleilat, Andrew Tawse-Smith, Sunyoung Ma, Lifeng Lin, Nabeel H. M. Alsabeeha

**Affiliations:** ^1^Mohammed Bin Rashid University of Medicine and Health Sciences, Hamdan Bin Mohammed College of Dental Medicine, Dubai Healthcare City, Dubai, UAE; ^2^Sir John Walsh Research Institute, Faculty of Dentistry, University of Otago, Dunedin, New Zealand; ^3^Department of Periodontology, Dubai Healthcare Authority, Dubai, UAE; ^4^Department of Epidemiology and Biostatistics, Mel and Enid Zuckerman College of Public Health, University of Arizona, Tucson, AZ, USA; ^5^Prosthetic Section, Ras Al-Khaimah Dental Center, Emirates Health Services, Dubai, UAE

## Abstract

**Methods:**

A retrospective analysis of patients aged ≥18 years and having dental implants placed at Dubai Health Authority in 2010. Relevant information related to systemic-, patient-, implant-, site-, surgical- and prosthesis-related factors were collected. The strength of association between the prevalence of peri-implant mucositis and peri-implantitis and each variable was measured by chi-square analysis. A binary logistic regression analysis was performed to identify possible risk factors.

**Results:**

A total of 162 patients with 301 implant-supported restorations were included in the study. The age of the patients ranged between 19 and 72 with a mean age of 46.4 ± 11.7 years. The prevalence of peri-implant mucositis at the patient and implant levels were 44.4% and 38.2%, respectively. For peri-implantitis, the prevalence at the patient level was 5.6%, while the prevalence at the implant level was 4.0%. The binary logistic regression identified three risk factors (smoking habits, histories of treated periodontitis and lack of peri-implant maintenance) for peri-implantitis.

**Conclusion:**

Within the limitations of this study, smoking habits, history of treated periodontitis and lack of peri-implant maintenance were significant risk factors for peri-implantitis. Early detection of these factors would ensure appropriate planning and care of patients at high risk of developing peri-implant diseases.

## 1. Introduction

In the replacement of missing teeth, treatment outcomes with fixed dental prostheses are associated with technical and biological complications in the long term. With dental implants, biological and technical complications were observed in 33.6% of patients during a 5-year follow-up period [1]. Biological complications of dental implants, (i.e. peri-implant mucositis and peri-implantitis) can negatively impact the longevity of dental implants leading to implant failure [2–4]. Peri-implant mucositis is defined as an inflammatory lesion of the mucosa surrounding a functionally osseointegrated dental implant while peri-implantitis is a progressive inflammatory disease affecting the peri-implant bone [5].

Peri-implant disease classification, case definition, and diagnostic criteria set to define peri-implant diseases, however, remain highly controversial [6–9]. These variations have made the assessment of the true prevalence of peri-implant diseases and associated risk factors very arduous [10, 11]. For example, a prevalence of 46.8% and 19.8% at the patient level were reported for peri-implant mucositis and peri-implantitis, respectively [12], while other reviews, estimated the equivalent at 64.3% and 18.8%, respectively [5, 13]. On the other hand, several risk factors were implicated in the onset and development of peri-implantitis [14]. For instance, patient-related risk factors such as inadequate plaque control, smoking, history of periodontitis, and lack of regular periodontal maintenance were identified in several retrospectives and cross-sectional studies [15]. Systemic-related risk factors such as diabetes mellitus [16] and factors related to implant design or surface characteristics were also described [17]. The need to identify risk factors associated with the onset and progression of peri-implant diseases for clinicians to set up effective preventive and maintenance regimens cannot be over-emphasized. These programs should be based on unequivocal case definitions and quality reporting of peri-implant disease prevalence [8]. The aims of the present study, therefore, were to evaluate the prevalence of peri-implant diseases and identify systemic-, patient-, implant-, site-, surgical- and prosthesis-related risk factors associated with the onset of peri-implant diseases.

## 2. Materials and Methods

### 2.1. Study Design and Participants

This was a retrospective study that included patients aged ≥18 years and having dental implants placed at Dubai Health Authority (DHA) in 2010. All the patients had periodontal and radiographic assessment prior to dental implant placement and during the follow-up visits up to 2019. The patients were advised to follow a regular maintenance program every six months with a hygienist. The patient records at DHA included demographic data, medical and dental history as well as the number of follow-up and maintenance visits. The current study was prepared in compliance with the Strengthening the Reporting of Observational Studies in Epidemiology (STROBE) guidelines [18].

### 2.2. Inclusion Criteria

The following are the criteria: aged ≥18 years; had dental implants placed at DHA in 2010; had sufficient record of clinical parameters and radiographic examination at each follow-up visit; had maintained a stable periodontal health during the follow-up period.

### 2.3. Exclusion Criteria

Records with missing data on more than 50% of the follow-up time were excluded.

### 2.4. Ethical Approval

The study was approved by the DHA and the institutional review board of Mohammed Bin Rashid University of Medicine and Health Sciences (MBRU-IRB-2020-014) in accordance with the Declaration of Helsinki of ethical human research practice.

### 2.5. Data Collection

Data were collected by one of the investigators (Z.A.) using dental practice management software (D4W, Australia), Salama software, and explanted implant records available at DHA. A standardized data collection form was used to collect relevant information. The data were collated into four main domains:

- Demographic data.
- Systemic and patient-related outcomes.
- Implant-, site-, and surgical-related outcomes.
- Prosthesis-related outcomes.

Patients were not recalled for examination. The information related to the clinical assessment, implant system, and radiographic marginal bone level changes were obtained from patient records. Data collection was divided into several sub-categories: systemic-related factors such as medical and social history, which were assessed at the time of implant placement. Implant-, surgical- and prosthesis-related factors such as implant system, location, surface roughness, height, diameter, shape, placement protocol, number of functional years prior to peri-implant diseases, use of grafting materials at the time of implant placement, type of retention, screw loosening, number of maintenance visits were also collected and analyzed as possible risk factors for peri-implantitis.

### 2.6. Systemic- and Patient-Related Factors

- Gender.
- Systemic conditions.
- Diabetes Mellitus.
- Dyslipidemia.
- Hypertension.
- Osteoporosis.
- Anemia.
- Hypothyroidism.
- Smoking habits.
- Parafunctional habits.
- History of treated periodontitis.
- Lack of regular dental attendance (regular attendance was defined as at least one dental check-up per year).
- Lack of regular peri-implant maintenance (regular maintenance was defined as at least one dental visit for peri-implant maintenance per year).

### 2.7. Implant- And Site-Related Factors

- Implant system (implant surface characteristics).
- Implant shape.
- Implant height.
- Implant diameter.
- Implant location.
- Implant placement protocols [19].
- Use of grafting material at the time of implant placement.
- Operator.

### 2.8. Prosthesis-Related Factors

- Type of prosthesis.
- Suprastructure retention.
- Number of functional years prior to diagnosis.
- Prosthetic complications: screw loosening.
- Prosthetic complications: crown chipping.
- Prosthetic complications: crown debonding.

Records with missing data on more than 50% of the follow-up time were excluded.

### 2.9. Case Definition

Peri-implant mucositis was defined as an osseointegrated functional implant that demonstrated bleeding and/or suppuration on probing, absence of increasing probing depths, and bone loss beyond initial remodeling of crestal bone levels. Peri-implantitis was defined as an osseointegrated functional implant that demonstrated bleeding and/or suppuration on probing, increased probing depths and bone loss beyond initial remodeling of crestal bone levels or >2 mm in the absence of baseline clinical parameters. A healthy implant was defined as one which showed no clinical signs of inflammation, absence of increased probing depths and bone loss beyond initial remodeling of crestal bone levels [7, 20].

### 2.10. Reliability Study

An experienced clinician (M.A.) conducted a training session on data collection which included running a practice exercise using a predetermined collection form from an actual patient file. To produce stable and consistent results, an intra-examiner reliability tests were performed by selecting five files from a pool of retrieved patient files. Data were collected by the investigator (Z.A.) two weeks apart and cross-checked by an experienced clinician (M.A.). The strength of intra-examiner reliability was assessed by calculating Cohen's kappa coefficients for selected items with two or more categories. Kappa scores of 0.21–0.40 indicated fair reliability; 0.41–0.60 indicated moderate reliability; 0.61–0.80 indicated substantial reliability; and 0.81–1.0 indicated excellent reliability [21].

### 2.11. Power Analysis

The determination of the sample size needed was based on adopting 95% power and 5% error using G*∗*power software (version 3.1.9.4). A representative sample size of 262 implants was calculated for the inclusion of at least 15 risk factors. To account for possible exclusions, a total of 300 implants were included.

### 2.12. Statistical Analyses

Data were analyzed using SAS statistical software version 9.4. The strength of association between the prevalence of peri-implant diseases and each variable was measured by exact chi-square analysis. Differences were considered statistically significant at *p* < 0.05. Estimates of relative risk were also calculated for all variables. For systemic and patient-related factors, the patient was considered the unit of analysis. Therefore, only one event of peri-implant mucositis or peri-implantitis per patient was included in the analysis to enhance statistical accuracy [22]. For implant-, site-, and prosthesis-related factors, the implant was considered as the statistical unit.

Risk factors for peri-implantitis were estimated by a binary logistic regression, which is the appropriate model for a categorical dichotomous outcome (peri-implantitis was coded 0 if no events occurred and 1 if peri-implantitis was reported). A backward stepwise method was selected. All predictor variables which had *p* values of less than 0.05 or a relative risk of 1.5 or greater were entered into the analysis and coded in a binary format of 0 or 1. Then, at each step, the variable with a significance level equal to or larger than 0.05 was removed, until the final model was obtained.

## 3. Results

A total of 162 patients with 301 implant-supported restorations were included in the study. The age of the patients ranged between 19 and 72 with a mean age of 46.4 ± 11.7 years. The prevalence of peri-implant mucositis at the patient and implant levels were 44.4% and 38.2%, respectively. For peri-implantitis, the prevalence at the patient level was 5.6%, while the prevalence at the implant level was 4.0%. The kappa values for the intra-examiner agreement ranged between 0.88 and 0.94, indicating excellent agreement in the data collection.

### 3.1. Peri-Implant Mucositis

Patients diagnosed with peri-implant mucositis were more likely to be irregular attenders of peri-implant maintenance visits as 55.4% of irregular attenders were diagnosed with peri-implant mucositis compared to 35.2% of regular attenders diagnosed with peri-implant mucositis (*p*=0.012). There was, however, no significant association between history of treated periodontitis or lack of regular dental attendance and peri-implant mucositis. Gender, presence of any systemic conditions, smoking and parafunctional habits had no significant impact on the prevalence of peri-implant mucositis (Table 1). All implants placed were roughened-surface Ankylos, Xive or Friadent implants with grit-blasted, acid-etched implant surfaces (Friadent plus, Dentsply Sirona), hence, it was not possible to compare the impact of implant surface characteristics on the prevalence of peri-implant mucositis. Cement-retained single crown implant restorations were more likely to be associated with an increased prevalence of peri-implant mucositis. However, the difference was not statistically significant when compared with screw-retained restorations. The implant dimension, implant location, implant placement protocol, use of grafting materials, operator or any prosthesis-related factors did not have any significant influence on the prevalence of peri-implant mucositis (Table 2).

### 3.2. Peri-Implantitis

The prevalence of peri-implantitis was statistically significant among smokers, those with a history of treated periodontitis and patients who did not attend regular peri-implant maintenance visits. Gender, presence of any systemic conditions, parafunctional habits, and lack of regular dental attendance had no significant impact on the prevalence of peri-implantitis (Table 3). Likewise, implant-, site-, surgical- and prosthesis-related factors did not have any significant influence on the prevalence of peri-implantitis (Table 4).

The binary logistic regression showed that smoking habits, history of treated periodontitis, and lack of peri-implant maintenance had a statistically significant association with the onset of peri-implantitis in the final model. The three variables had low standard errors implying a statistically stable model and did not contain a value of 1.00 representing useful and independent predictor variables. The odds ratios showed that smokers, those with a history of treated periodontitis, and those who did not attend regular peri-implant maintenance were eight, seven, and ten times at risk of peri-implantitis, respectively. The overall accuracy of the model to predict peri-implantitis (with a predicted probability of 0.5 or greater) was 96.3%. The estimates of the logistic regression model, the adjusted odds ratios for the three risk factors and their 95% CIs are summarized in Table 5.

## 4. Discussion

The present study reports on the prevalence rates of peri-implant mucositis and peri-implantitis and associated risk factors over a 10-year follow-up period. The prevalence rates for peri-implant mucositis were 44.4% and 38.0% at patient and implant levels, respectively. The corresponding rates for peri-implantitis were 5.6% and 4.0% at patient and implant levels, respectively.

The prevalence of peri-implant mucositis reported in the present study was higher than previously reported [23] where the estimated prevalence of peri-implant mucositis was 20.2% at the patient level and 10.2% at the implant level. The present results, however, were comparable with other studies [24, 25]. In one study [25] with a total of 4591 implants and a follow-up period of 5 to 10 years, the estimated prevalence of peri-implant mucositis was 38.6% at the implant level, while that of peri-implantitis was 4.7%. Similarly, a cross-sectional study of 24 of 96 patients with 225 implants and a follow-up period of 11 years reported a prevalence rate of 48% for peri-implant mucositis at the patient level and 33% at the implant level. On the other hand, the prevalence rate for peri-implantitis reported in the present study was higher than those reported previously, at both the patient and implant levels. The observed differences in results amongst the different studies could be a reflection of the differences in the study designs and the clinical and radiographic criteria used for diagnosing peri-implant diseases. It is well documented that using different thresholds of severity in reporting prevalence of peri-implantitis results in a wide range of peri-implantitis prevalence rates [10, 26]. In this context, our study adopted the definition of the 2017 World Workshop [7, 20] to allow comparability with other future studies.

### 4.1. Systemic- And Patient-Related Factors

The present review has shown that lack of regular peri-implant maintenance was a significant risk factor for the development of peri-implant diseases as patients who did not undergo regular peri-implant maintenance were at ten times higher risk of peri-implantitis. This significant association is in accordance with the findings of other studies [13, 23, 27, 28]. In one study of 212 participants [27], the prevalence of peri-implantitis amongst patients without supportive peri-implant care was 43.9% compared to 18.0% in those that followed regular peri-implant maintenance.

History of treated periodontitis was another significant factor associated with peri-implantitis in the present study. Patients with a history of treated periodontitis were found to be at a seven times higher risk of peri-implantitis than periodontally healthy patients. Similar findings were reported in several long-term studies [26, 28–37]. With over a 10-year follow-up period, Karoussis and co-workers [38] showed that the prevalence of peri-implantitis for periodontally compromised and healthy patients were 28.6% and 5.8%, respectively. A systematic review of 24 longitudinal studies highlighted the high risk for peri-implantitis in patients with treated periodontal diseases [39]. Besides the history of treated periodontitis, recurrent periodontal disease with residual probing depths of six or more millimeters during follow-ups has shown to have a significant impact on the incidence of peri-implantitis [40]. In another study of 70 periodontally-treated patients with 165 dental implants who were followed up for 3 to 23 years, the risk for developing peri-implantitis was significantly high in patients with a history of periodontitis and residual probing depths of ≥5 mm 28.

The third risk factor found to be significant in this study was smoking with smokers having eight times higher risk for peri-implantitis than non-smokers. This was in accordance with several studies and systematic reviews [13, 15, 23, 41, 42]. In one systematic review [13], smokers had a prevalence of peri-implantitis of 36.3%. Another systematic review and meta-analysis of 19,836 implants showed a higher incidence of postoperative infections and implant failure amongst smokers compared with non-smokers [42]. Smokers were also repeatedly reported to have a significant increase in peri-implant bone loss over time [43–45]. In addition to signs of inflammation, progressive bone loss beyond the bone level changes of initial remodeling was another observation that accompanied the diagnosis of peri-implantitis.

### 4.2. Implant-And Site-Related Risk Factors

An association between implant surface characteristics and risk for peri-implant diseases could not be detected since the implants used in this study were roughened-surface implants. Significant peri-implant marginal bone loss was observed around plasma-sprayed titanium implants compared to minimally roughened implants [46]. However; other studies [17, 23, 47] failed to demonstrate implant system characteristics as an independent risk factor for peri-implantitis.

The present study showed that implant placement protocol and use of grafting materials did not have any significant influence on the prevalence of peri-implant diseases. In contrast, a retrospective analysis of 188 patients with 423 implant-supported restorations showed that immediate implant placement and use of grafting material were significantly associated with peri-implant mucositis but were not identified as risk factors for peri-implantitis [23]. Interestingly, a retrospective study with a large sample size of 1017 patients and 3082 implant-supported restorations showed that the use of non-autogenous bone grafting material with immediate implant placement increased the risk of early inflammation [48]. The lack of correlation between implant placement protocol or use of grafting materials and risk for peri-implant diseases; however, it does not necessarily exclude these variables as potential risk factors for early inflammation and peri-implant disease. In the present study, the limited number of immediately placed implants or cases where bone grafting was used may not have allowed proper assessment of these variables as risk factors.

### 4.3. Prosthesis-Related Risk Factors

There was no significant association between any of the prosthesis-related factors evaluated in this study and an increase in the risk for peri-implant diseases. This does not preclude that some prosthesis-related factors could be identified as potential risk factors. For example, cement-retained implant restorations were often accompanied by signs of peri-implant inflammation. Complete removal of subgingival excess cement may not be possible with a subsequent predisposition to peri-implant marginal bone loss and peri-implantitis [49]. A retrospective study of 249 implants has shown that cement-retained implant restorations had a 4.6 times higher risk of gingival inflammation compared to screw-retained implant restorations [50]. However, a statistically significant difference between cement- and screw-retained restorations was not found in terms of implant survival despite reporting more incidents of biological complications around cement-retained restorations [51, 52].

In the present study, a higher prevalence of peri-implantitis was reported around cement-retained implant restorations compared to screw-retained ones but the difference was not statistically significant. Placement of restoration margins closer to the most coronal mucosal levels might have allowed early detection and ease of removal of excess cement. Nevertheless, this cannot be confirmed as none of the participants were recalled to clinically assess the position of the restoration margins.

Several limitations can be identified in the present study. The retrospective design of the study with the inherent difficulty in collecting data or controlling all confounding factors is common. In addition, some outcomes were reported by a small number of cases and this might have underpowered the study. The use of implants from three systems could limit the extrapolation of the present study findings. Nevertheless, all included implants were placed in a standard clinical setting using a standardized surgical approach which might have reduced the impact of other confounding factors. In addition, the present study reported a nine-year follow-up where all the implants were placed in 2010, and data was collected up to 2019.

## 5. Conclusions

Within the limitations of this study, smoking habits, history of treated periodontitis and lack of peri-implant maintenance were significant risk factors for peri-implantitis. Early detection of these factors would ensure appropriate planning and care of patients at high risk of developing peri-implant diseases. Further studies with larger sample sizes are still required particularly in relation to the outcomes where data was not sufficient.

## Figures and Tables

**Table 1:** Characteristics of patients diagnosed with peri-implant mucositis (*n* = 162).

Systemic and patient-related factors:	*N* (%) diagnosed peri-implant mucositis	Relative risk (95% CI)	*p* value^*∗*^
Gender		1.34 (0.95, 1.88)	0.109
Male	34 (52.3)		
Female	38 (39.2)		

Presence of systemic conditions		1.04 (0.72, 1.52)	0.866
Yes	22 (43.1)		
No	50 (45.0)		

Presence of diabetes mellitus		0.83 (0.55, 1.24)	0.415
Yes	15 (51.7)		
No	57 (42.9)		

Presence of dyslipidemia		0.88 (0.50, 1.53)	0.781
Yes	7 (50.0)		
No	65 (43.9)		

Presence of hypertension		1.03 (0.62, 1.70)	1.000
Yes	10 (43.5)		
No	62 (44.6)		

Presence of osteoporosis		3.21 (0.52, 19.84)	0.133
Yes	1 (14.3)		
No	71 (45.8)		

Presence of anemia		0.89 (0.33, 2.40)	1.000
Yes	2 (50.0)		
No	70 (44.3)		

Presence of hypothyroidism		1.20 (0.48, 2.97)	0.734
Yes	3 (37.5)		
No	69 (44.8)		

Smoking habits		1.01 (0.61, 1.67)	1.000
Smokers	10 (43.5)		
Non-smokers	60 (43.8)		

Parafunctional habits		2.55 (0.72, 9.04)	0.114
Yes	2 (18.2)		
No	70 (46.4)		

History of treated periodontitis		0.85 (0.59, 1.24)	0.466
Yes	20 (50.0)		
No	52 (42.6)		

Lack of regular dental attendance		1.31 (0.89, 1.92)	0.221
Yes	16 (55.2)		
No	56 (42.1)		

Lack of regular peri-implant maintenance		1.57 (1.11, 2.23)	0.012
Yes	41 (55.4)		
No	31 (35.2)		

CI: confidence interval. ^†^Exact chi-square test.

**Table 2:** Characteristics of implants diagnosed with peri-implant mucositis (*n* = 301).

Implant-, site-, and surgical-related outcomes	*N* (%) diagnosed peri-implant mucositis	Relative risk (95% CI)	*p* value^*∗*^
Implant system		NA	0.458
Ankylos	48 (38.7)		
Xive	66 (39.1)		
Friadent	0 (0.0)		

Implant shape		1.01 (0.75, 1.35)	1.000
Cylindrical	48 (38.7)		
Tapered	66 (38.4)		

Implant height (mm)		0.97 (0.72, 1.30)	0.903
<11	72 (38.9)		
≥11	43 (37.7)		

Implant diameter (mm)		0.95 (0.71, 1.27)	0.722
<4.5	65 (39.4)		
≥4.5	50 (37.3)		

Implant location		NA	0.308
Anterior maxilla	8 (25.0)		
Posterior maxilla	40 (36.7)		
Anterior mandible	4 (50.0)		
Posterior mandible	63 (41.4)		

Implant placement protocol		NA	0.580
Type I	7 (43.8)		
Type II	3 (30.0)		
Type III	6 (26.1)		
Type IV	99 (39.3)		

Bone augmentation procedure at the time of implant placement		1.14 (0.78, 1.67)	0.50
Yes	21 (34.4)		
No	94 (39.2)		

Operator		0.89 (0.66, 1.21)	0.463
Periodontist	76 (39.8)		
Oral and maxillofacial surgeon	39 (35.5)		
			

Prosthesis-related outcomes	*N* (%) diagnosed peri-implant mucositis	Relative risk (95% CI)	*p* value^*∗*^

Type of prosthesis		1.34 (0.89, 2.01)	0.148
Single implant crown	96 (40.3)		
Multiple-unit implant-supported prosthesis	19 (30.2)		

Superstructure retention		0.91 (0.67, 1.23)	0.538
Screw-retained	40 (35.7)		
Cement-retained	71 (39.4)		

Number of functional years prior to diagnosis		1.26 (0.95, 1.68)	0.123
<5 years	59 (43.1)		
≥5 years	56 (34.1)		

Prosthetic complications		0.93 (0.58, 1.50)	0.837
Screw loosening			
Yes	11 (40.7)		
No	104 (38.0)		

Prosthetic complications		1.15 (0.56, 2.40)	0.790
Crown chipping			
Yes	5 (33.3)		
No	110 (38.5)		

Prosthetic complications		1.00 (0.67, 1.48)	1.000
Crown debonding			
Yes	18 (38.3)		
No	97 (38.2)		

CI: confidence interval. ^*∗*^Exact chi-square test.

**Table 3:** Characteristics of patients diagnosed with peri-implantitis (*n* = 162).

Systemic and patient-related factors:	*N* (%) diagnosed peri-implantitis	Relative risk (95% CI)	*p* value^*∗*^
Gender		0.43 (0.09, 1.99)	0.317
Male	2 (3.1)		
Female	7 (7.2)		

Presence of systemic conditions		0.92 (0.24, 3.53)	1.000
Yes	3 (5.9)		
No	6 (5.4)		

Presence of diabetes mellitus		0.76 (0.17, 3.49)	1.000
Yes	2 (6.9)		
No	7 (5.3)		

Presence of dyslipidemia		0.76 (0.10, 5.62)	1.000
Yes	1 (7.1)		
No	8 (5.4)		

Presence of hypertension		1.32 (0.17, 10.09)	1.000
Yes	1 (4.3)		
No	8 (5.8)		

Presence of osteoporosis		0.36 (0.05, 2.50)	0.335
Yes	1 (14.3)		
No	8 (5.2)		

Presence of anemia		0.20 (0.03, 1.26)	0.206
Yes	1 (25.0)		
No	8 (5.1)		

Presence of hypothyroidism		0.42 (0.06, 2.93)	0.374
Yes	1 (12.5)		
No	8 (5.2)		

Smoking habits		9.24 (2.26, 37.60)	0.003
Smokers	5 (21.7)		
Non-smokers	4 (2.9)		

Parafunctional habits		0.58 (0.08, 4.25)	1.000
Yes	1 (9.1)		
No	8 (5.3)		

History of treated periodontitis		7.00 (1.66, 29.47)	0.008
Yes	6 (15.0)		
No	3 (2.5)		

Lack of regular dental attendance		0.57 (0.07, 4.41)	0.701
Yes	1 (3.4)		
No	8 (6.0)		

Lack of regular peri-implant maintenance		9.51 (1.22, 74.33)	0.012
Yes	8 (10.8)		
No	1 (1.1)		

CI: confidence interval. ^*∗*^Exact chi-square test.

**Table 4:** Characteristics of implants diagnosed with peri-implantitis (*n* = 301).

Implant-, site-, and surgical-related outcomes	*N* (%) diagnosed peri-implantitis	Relative risk (95% CI)	*p* value^*∗*^
Implant system			
Ankylos	6 (3.6)	NA	0.794
Xive	6 (4.8)		
Friadent	0 (0.0)		

Implant shape		1.39 (0.46, 4.20)	0.767
Cylindrical	6 (4.8)		
Tapered	6 (3.5)		

Implant height (mm)		1.62 (0.54, 4.91)	0.546
<11	6 (3.2)		
≥11	6 (5.3)		

Implant diameter (mm)		0.41 (0.11, 1.49)	0.237
<4.5	9 (5.5)		
≥4.5	3 (2.2)		

Implant location		NA	0.070
Anterior maxilla	1 (3.1)		
Posterior maxilla	3 (2.8)		
Anterior mandible	0 (0.0)		
Posterior mandible	8 (5.3)		

Implant placement protocol			
Type I	0 (0.0)	NA	0.906
Type II	0 (0.0)		
Type III	1 (4.3)		
Type IV	11 (4.4)		

Bone augmentation procedure at the time of implant placement		1.27 (0.29, 5.65)	0.75
Yes	2 (3.3)		
No	10 (4.2)		

Operator		2.43 (0.79, 7.48)	0.131
Periodontist	5 (2.6)		
Oral and maxillofacial surgeon	7 (6.4)		

Prosthesis-related outcomes	*N* (%) diagnosed peri-implant mucositis	Relative risk (95% CI)	*p* value^*∗*^

Type of prosthesis		0.79 (0.22, 2.85)	1.000
Single implant crown	9 (3.8)		
Multiple-unit implant-supported prosthesis	3 (4.8)		

Superstructure retention		1.15 (0.37, 3.53)	1.000
Screw-retained	5 (4.5)		
Cement-retained	7 (3.9)		

Number of functional years prior to diagnosis		2.39 (0.74, 7.78)	0.150
<5 years	8 (5.8)		
≥5 years	4 (2.4)		

Prosthetic complications		1.08 (0.15, 8.08)	1.000
Screw loosening			
Yes	1 (3.7)		
No	11 (4.0)		

Prosthetic complications		1.79 (0.22, 14.82)	1.000
Crown chipping			
Yes	1 (6.7)		
No	11 (3.8)		

Prosthetic complications		1.08 (0.23, 5.12)	1.000
Crown debonding			
Yes	2 (4.3)		
No	10 (3.9)		

CI: confidence interval. ^*∗*^Exact chi-square test.

**Table 5:** Results of logistic regression analysis.

Predictor variable	Peri-implantitis		
	B Coefficient (SE)	*p* Value	Odds ratio (95% CI)
Smoking habits (yes = 0, no = 1)	2.15	0.008	8.55 (1.75, 42.10)
History of treated periodontitis (yes = 0, no = 1)	1.98	0.014	7.26 (1.48, 35.52)
Lack of regular peri-implant maintenance (yes = 0, no = 1)	2.34	0.036	10.41 (1.15, 93.69)

Model Chi-square = 22.88; *d*f = 3; *p* < 0.0001. CI: confidence interval.

## Data Availability

The data that support the findings of this study are available on request from the corresponding author. The data are not publicly available due to privacy or ethical approval.

## Additional Points

Clinical Relevance. *Scientific Rationale for the Study*. There is an indication that the prevalence of peri-implant diseases has increased over the last two decades. However, there is still a lack of literature on risk factors for peri-implant diseases in specific populations. *Principal Findings*. Smokers, those with a history of treated periodontitis, and patients who did not attend regular peri-implant maintenance visits were at high risk of developing peri-implantitis. *Practical Implications*. Early detection of smoking habits, history of treated periodontitis and lack of peri-implant maintenance may help clinicians in developing individualized preventive management strategies to maintain the longevity of dental implants.
